# Intra-Day and Inter-Day Reliability of Measurements of the electromyographic signal on masseter and temporal muscles in patients with Down syndrome

**DOI:** 10.1038/s41598-020-63963-z

**Published:** 2020-05-04

**Authors:** Lilian Chrystiane Giannasi, Fabiano Politti, Marignês T. S. Dutra, Vera L. S. Tenguan, Gabriela R. C. Silva, Gabriela P. Mancilha, Daniel Batista da Silva, Luis Vicente Franco Oliveira, Claudia Santos Oliveira, Jose B. O. Amorim, Miguel Angel Castillo Salgado, Mônica F. Gomes

**Affiliations:** 1 Center of Biosciences Applied to Patients with Special Health Care Needs (CEBAPE), Institute of Science and Technology, São José dos Campos Campus, São Paulo State University–UNESP, São Paulo, SP Brazil; 2 Nove de Julho University, São Paulo, Brazil; 3 Centro Universitário de Anápolis - UniEvangélica, São Paulo, Brazil

**Keywords:** Electromyography - EMG, Rehabilitation

## Abstract

The aim of the present study was to evaluate intra-day (test) and inter-day (re-test) reliability of surface electromyography (sEMG) signals of the masseter and temporal muscles in patients with Down syndrome (DS). We determined the reliability of sEMG variables in 33 patients with DS. EMG signals were recorded at rest as well as during maximum voluntary clenching and maximum habitual intercuspation (MHI). The signals were analyzed considering the amplitude in the root mean square (RMS), mean frequency (MNF), median frequency (MDF) and approximate entropy (ApEn). The intraclass correlation (ICC_2,1_) for the three trials recorded during MHI in the two sessions (test and retest) revealed excellent intra-session and inter-session reliability (ICC_2,1_ = 0.76 to 0.97) for all sEMG variables and muscles. In the rest position, excellent reliability was found for RMS and ApEn (ICC_2,1_ = 0.75 to 1.00) and good to excellent reliability was found for MDF and MNF (ICC_2,1_ = 0.64 to 0.93). The intra-session (test) and inter-session (re-test) analyses demonstrated the reliability of nonlinear sEMG variables of the masticatory muscles in adults with Down Syndrome.

## Introduction

Down syndrome (DS), also known as trisomy of chromosome 21 (HSA21), is the most common genetic alteration, the prevalence of which ranges from six to 13 per every 10000 people in the general populatio^1,2^. This condition is associated with cognitive impairment, several comorbidities and emotional-social limitations, leading to substantial medical and social costs^3–6^.

Generalized muscular hypotonia is one of characteristics of DS, which, along with oral abnormalities, directly affects oral functions, such as swallowing, speech and breathing (even during sleep, leading to obstructive sleep apnea)^7^. The combination of abnormal masticatory muscle function (due to hypotonia) and altered skeletal developmental (e.g., discrepancy between alveolar arches, reduced maxillary length and midface retrusion) has several consequences during the growth phase, such as oromotor incoordination (weak jaw-closing muscles, hypotonicity of the tongue and inefficient lingual lateralization), difficulties during meals (choking, belching and food spillage from the mouth), uncontrolled facial movements, mouth open at rest and mouth-breathing due to poor muscle tonicity^8–10^.

Some of these abnormal muscle functions, such as those in the muscles responsible for the sustaining and moving the jaw (masseter and temporal) can be measured using surface electromyography (EMGs). However, the selection of a measure for research or clinical use depends on several factors, such as reliability. In clinical practice, the combination of linear and nonlinear measures is important to the characterization, classification and evaluation of the complexity of sEMG variables of the masticatory muscles and can enable a better understanding of neurophysiological conditions.

The root mean square (RMS) is a linear variable used to evaluate the excitability and activation of muscles before and after therapy^11,12^. Median frequency (MDF) is another linear variable widely used to interpret spectral characteristics of sEMG signals, reflecting muscle electrophysiology in different clinical conditions such as temporomandibular disordes^13^, cerebral palsy^14^ and low back pain^15^.

Nonlinear time-series analyses indicate features of motor control that are important for clinicians to measure^16^. Information entropy has been proposed as a measure of irregularity (nonlinear behavior) in biological signals^16–19^. The measurement of entropy is reported to be a reliable method for characterizing neuromuscular changes^20^ and approximate entropy (ApEn) enables a general understanding of the complexity of sEMG data^21,22^. In statistics, ApEn is used to establish the uncertainty or variability in a system. ApEn calculated from sEMG signals is dependent on the variability in both amplitude and frequency and better represents this aspect than amplitude or frequency alone^21,22^. As studies have demonstrated that nonlinear time-series analysis is more sensitive to changes in myoelectrical signals in comparison to linear methods^23,24^, the use of ApEn to quantify the irregularity or complexity of sEMG signals of the masseter and anterior temporal muscles in patients with DS may be important to the quantification and understanding of the neurophysiological conditions of these muscles.

However, the usefulness of sEMG data is dependent on the reproducibility of the signal detection method both within and between recording sessions^25^. This is a key factor to evaluating sEMG data in rehabilitation programs and clinical trials. Factors such as the preparation of the skin and positioning of the electrodes can exert an influence on the results when sEMG data are collected on different days^26,27^, as occurs when signals are collected before and after a clinical intervention.

Although a number of studies have demonstrated the reliability and reproducibility of sEMG signals for the evaluation of the masticatory muscles in both healthy and disabled patients^17,21,28^, to the best of our knowledge, no studies have assessed the reliability of sEMG signals of the masticatory muscles or the reproducibility of sEMG variables (linear and nonlinear) of the masseter and anterior temporal muscles in adults with DS. It is important to determine the reproducibility of measuring sEMG signals (intra-session and inter-session reliability) prior to suggesting the use of this technique as a tool for evaluating the efficacy of therapy designed to improve masticatory muscle function in this population.

Therefore, the aim of the present study was to determine the intra-session (test) and inter-session (re-test) reliability of sEMG signals of the masseter and anterior temporal muscles in adults with DS.

## Results

The sample was composed of 23 adults with DS (15 men and eight women) with a mean age of 22.7 ± 6.5 years, mean body mass index (BMI) of 28.5 ± 6.8 kg/m^2^ and mean neck circumference of 40.3 ± 4.2 cm (Table 1).

**Table 1:** Demographic and anthropometric data of 23 Down Syndrome patients.

Variables	Baseline mean/SD
Age (years)	22.7 ± 6.5
Gender (female/male)	8/15
BMI (Kg/cm^2^)	28.5 ± 6.8
Neck circumference (cm)	40.3 ± 4.2
Abdomen circumference (cm)	93.7 ± 16.5

Note: BMI = body mass index.

The reliability of the sEMG variables was determined in all participants. Tables 2 and 3 displays the mean (SD), ICC and SEM values for each sEMG measurement during MIH and in the rest position for each muscle. The ICCs for all sEMG parameters and muscles during MHI in the two sessions (test and retest) revealed excellent intra-session and inter-session reliability (range: 0.76 to 0.97) (Table 2). In the rest position, excellent reliability was found for RMS and ApEn (range: 0.75 to 1.00), whereas good to excellent reliability was found for MDF and MFN (range: 0.64 to 0.93).

**Table 2:** Intra-day (Test and Retest) and between-day (Test/Retest) reliability of EMG signal measurements in MHI position.

	Test			Retest			Test/Retest		
	Mean (SD)	ICC_2,1_ (95%CI)	SEM	Mean (SD)	ICC_2,1_ (95%CI)	SEM	Mean (SD)	ICC_2,1_ (95%CI)	SEM
RMS MHI									
RT	57.88 (33.26)	0.97 (0.93 to 0.99)	5.73	57.59 (36.46)	0.76 (0.51 to 0.89)	30.05	3.17 (3.81)	0.67 (0.36 to 0.85)	21.25
RM	57.55 (25.99)	0.90 (0.78 to 0.96)	8.27	57.27 (26.54)	0.84 (0.66 to 0.93)	10.60	2.92 (3.61)	0.78 (0.55 to 0.90)	12.28
LT	44.26 (32.79)	0.91 (0.90 to 0.96)	10.04	41.26 (26.26)	0.93 (0.84 to 0.97)	6.99	2.73 (2.32)	0.89 (0.76 to 0.95)	9.87
LM	48.29 (30.65)	0.86 (0.70 to 0.94)	11.45	48.56 (26.33)	0.85 (0.68 to 0.93)	10.14	3.64 (2.97)	0.82 (0.62 to 0.92)	11.99
FDM MHI									
RT	164.82 (40.40)	0.93 (0.84 to 0.97)	10.31	165.49 (42.50)	0.95 (0.89 to 0.98)	9.00	71.82 (26.49)	0.95 (0.89 to 0.98)	8.97
RM	155.82 (39.24)	0.96 (0.91 to 0.98)	8.17	162.10 (38.18)	0.89 (0.76 to 0.95)	12.35	87.58 (26.84)	0.95 (0.89 to 0.98)	8.88
LT	119.14 (40.73)	0.92 (0.82 to 0.97)	11.77	124.54 (42.73)	0.95 (0.89 to 0.98)	10.35	72.01 (31.24)	0.94 (0.85 to 0.97)	10.53
LM	118.46 (43.44)	0.94 (0.86 to 0.97)	10.89	125.90 (40.78)	0.93 (0.84 to 0.97)	10.75	34.97 (29.99)	0.94 (0.86 to 0.97)	10.21
FM MHI									
RT	182.56 (37.77)	0.95 (0.89 to 0.98)	8.63	181.69 (40.08)	0.97 (0.92 to 0.99)	7.33	105.28 (30.85)	0.96 (0.91 to 0.98)	7.59
RM	177.32 (36.90)	0.95 (0.88 to 0.98)	8.31	180.58 (36.27)	0.91 (0.90 to 0.96)	11.00	132.41 (37.19)	0.95 (0.89 to 0.98)	8.36
LT	139.66 (42.90)	0.94 (0.86 to 0.97)	10.48	148.83 (46.37)	0.95 (0.89 to 0.98)	10.69	116.33 (31.25)	0.94 (0.86 to 0.97)	10.41
LM	138.88 (38.68)	0.95 (0.89 to 0.98)	8.85	143.39 (40.39)	0.94 (0.86 to 0.97)	9.48	65.43 (4.83)	0.95 (0.89 to 0.98)	8.53
ApEn MHI									
RT	1.20 (0.20)	0.86 (0.70 to 0.94)	0.08	1.20 (0.23)	0.85 (0.68 to 0.93)	0.09	1.39 (0.25)	0.89 (0.76 to 0.95)	0.07
RM	1.20 (0.19)	0.82 (0.62 to 0.92)	0.08	1.24 (0.20)	0.78 (0.55 to 0.90)	0.13	1.48 (0.28)	0.83 (0.64 to 0.92)	0.08
LT	1.09 (0.28)	0.88 (0.74 to 0.95)	0.10	1.15 (0.26)	0.89 (0.76 to 0.95)	0.09	1.60 (0.22)	0.88 (0.74 to 0.95)	0.10
LM	1.10 (0.22)	0.88 (0.74 to 0.95)	0.08	1.08 (0.24)	0.87 (0.77 to 0.94)	0.09	1.38 (0.22)	0.90 (0.77 to 0.96)	0.07

ApEn: approximate entropy. EMG: electromyographic. ICC: intraclass correlation coefficient. LT: left temporal. LM: left masseter. MHI: maximum habitual intercuspation. MVC: maximal voluntary contraction. MDF: median frequency. MNF: mean frequency. RT: right temporal. RM: right masseter. RMS: root mean square. SEM: standard error of measurement. ZC: zero crossing. 95%CI: 95% confidence interval of the mean.

**Table 3:** Intra-day (Test and Retest) and between-day (Test/Retest) reliability of EMG signal measurements in rest position.

	Test			Retest			Inter day		
	Mean (SD)	ICC_2,1_ (95%CI)	SEM	Mean (SD)	ICC_2,1_ (95%CI)	SEM	Mean (SD)	ICC	SEM
RMS_rest									
RT	3.31 (5.25)	0.99 (0.98 to 1.00)	0.41	3.03 (2.38)	0.98 (0.95 to 0.99)	0.35	3.17 (3.81)	0.95 (0.89 to 0.98)	0.91
RM	2.28 (1.19)	0.83 (0.64 to 0.92)	0.50	3.57 (6.04)	0.99 (0.98 to 1.00)	0.31	2.925 (3.61)	0.88 (0.74 to 0.95)	1.52
LT	2.96 (2.50)	0.87 (0.77 to 0.94)	0.89	2.51 (2.15)	0.99 (0.98 to 1.00)	0.23	2.73 (2.32)	0.95 (0.89 to 0.98)	0.53
LM	3.51 (2.39)	0.80 (0.58 to 0.91)	1.10	3.77 (3.56)	0.99 (0.98 to 1.00)	0.15	3.64 (2.97)	0.91 (0.89 to 0.96)	0.89
FDM rest									
RT	69.18 (25.41)	0.80 (0.58 to 0.91)	11.59	74.46 (27.57)	0.86 (0.70 to 0.94)	10.69	71.82 (26.49)	0.66 (0.35 to 0.84)	15.97
RM	93.86 (26.07)	0.64 (0.32 to 0.83)	16.00	81.31 (27.62)	0.90 (0.78 to 0.96)	8.66	87.58 (26.84)	0.77 (0.53 to 0.90)	13.24
LT	72.29 (28.21)	0.85 (0.68 to 0.93)	11.16	71.74 (34.28)	0.89 (0.76 to 0.95)	11.30	72.01 (31.24)	0.77 (0.53 to 0.90)	14.95
LM	37.30 (29.84)	0.76 (0.51 to 0.89)	15.54	32.65 (30.15)	0.86 (0.69 to 0.94)	11.32	34.97 (29.99)	0.69 (0.40 to 0.86)	16.69
FM rest									
RT	105.89 (29.29)	0.88 (0.74 to 0.95)	10.17	104.67 (32.42)	0.91 (0.90 to 0.96)	10.12	105.28 (30.85)	0.83 (0.64 to 0.92)	12.91
RM	137.29 (35.92)	0.82 (0.62 to 0.92)	15.38	127.53 (38.47)	0.93 (0.84 to 0.97)		132.41 (37.19)	0.75 (0.50 to 0.89)	19.02
LT	115.18 (27.87)	0.76 (0.51 to 0.89)	14.03	117.48 (34.63)	0.90 (0.78 to 0.96)	10.85	116.33 (31.25)	0.69 (0.40 to 0.86)	17.45
LM	64.28 (22.20)	0.68 (0.38 to 0.85)	12.60	66.59 (27.46)	0.90 (0.78 to 0.96)	8.90	65.43 (4.83)	0.75 (0.50 to 0.89)	12.39
ApEn rest									
RT	1.40 (0.22)	0.83 (0.63 to 0.92)	0.09	1.38 (0.28)	0.91 (0.90 to 0.96)	0.08	1.39 (0.25)	0.83 (0.63 to 0.92)	0.10
RM	1.49 (0.28)	0.86 (0.70 to 0.94)	0.11	1.47 (0.29)	0.89 (0.76 to 0.95)	0.09	1.48 (0.28)	0.76 (0.51 to 0.89)	0.17
LT	1.59 (0.21)	0.75 (0.50 to 0.89)	0.11	1.62 (0.24)	0.89 (0.76 to 0.95)	0.08	1.60 (0.22)	0.82 (0.62 to 0.92)	0.10
LM	1.35 (0.18)	0.91 (0.90 to 0.96)	0.05	1.42 (0.27)	0.92 (0.82 to 0.97)	0.08	1.38 (0.22)	0.84 (0.66 to 0.93)	0.09

ApEn: approximate entropy. EMG: electromyographic. ICC: intraclass correlation coefficient. LT: left temporal. LM: left masseter. MHI: maximum habitual intercuspation. MVC: maximal voluntary contraction. MDF: median frequency. MNF: mean frequency. RT: right temporal. RM: right masseter. RMS: root mean square. SEM: standard error of measurement. ZC: zero crossing. 95%CI: 95% confidence interval of the mean.

## Discussion

The present study confirmed the reliability (calculated using the ICC and SEM) of different sEMG variables of the masseter and anterior temporal muscles recorded during MHI and in the rest position in adults with DS. The results of the linear (amplitude and frequency) and non-linear (ApEn) analyses revealed excellent intra-session and inter-session reliability of sEMG signal readings during MIH (Table 2) as well as good to excellent reliability in the rest position (Table 3).

These results are similar to those reported in a previous study involving patients with cerebral palsy, i.e., high reproducibility for the data recorded during MIH and good to excellent reproducibility for data recorded in the rest position^17^. A high degree of reliability during MHI has also been found for the anterior temporal and masseter muscles in healthy patients^29,30^. These results demonstrate that sEMG activity recorded during MIH and at rest can be used to quantify the effects of clinical interventions on the masseter and anterior temporal muscles.

The sEMG evaluation is diagnostically useful in identifying patients with pain-related temporomandibular disorders^31,32^ or for the observation of muscle activity in the clinical setting^30^. Multiparameter sEMG analysis has been used as a strategy to investigate the performance of the masticatory muscles following a stroke^33,34^, assess pain patterns in the masticatory muscles^35^ and investigate the reproducibility of the evaluation of the masticatory muscles in patients with cerebral palsy^17^.

Electromyographic components calculated in the time and frequency domains are the most widely used variables for the study of masseter and anterior temporal muscle activity, whereas nonlinear components, such as ApEn, remain under-investigated in clinical studies. This is an issue that needs to be better explored, as a change in the entropy of the signal also reflects a qualitative change in the physiological system^22^. Specifically for ApEn, the result is given between 0 and 2, with higher values reflecting greater irregularity within the time-series^36–38^. Thus, values near 2 indicate healthy muscle function. The excellent reliability found for ApEn under both test conditions (MIH and rest) could be an important stimulus for future studies to use this sEMG parameter in the evaluation of the effects of therapeutic interventions performed on the masseter and anterior temporal muscles.

## Conclusion

The present findings demonstrate the excellent reliability of surface electromyography variables (root mean squared, mean frequency, median frequency and approximate entropy) of the masseter and anterior temporal muscles in adults with Down Syndrome during maximum clenching effort as well as good to excellent reliability of the median and mean frequency recorded in the rest position.

## Materials and Methods

### Subjects

A convenience sample of adult patients with Down syndrome were evaluated at the Center of Bioscience Applied to Persons with Special Care Needs (CEBAPE), Institute of Science and Technology – UNESP/SJC, Brazil. The inclusion criteria were adult patients aged between 18–35 years-old, presenting snoring and/or mild to moderate apnea/hypopnea index, preserved cognitive function (ability to understand and respond to verbal commands necessary to perform this study, such as “open the mouth”, “close the mouth”, “ bite”, “relax”), agreed to participate by free will and, the Awareness Term along with signed by patient or patient’s responsible. The exclusion criteria were body mass index (BMI) greater than 30, unsatisfactory dental health, not able to reach the University when is needed, present psychiatric disease and have been submitted to physiotherapy, speech therapy and/or orthodontic treatment at least 6 months before the beginning of this study.

This study received approval from the Human Research Ethics Committees of the Institute of Science and Technology– UNESP/SJC, Brazil, CEPh 2.127.141 (process number CAAE 64173616.4.0000.0077).

### Instrumentation

The device used for the EMG acquisition was an eight-channel module (EMG System do Brasil Ltda®) consisting of a conditioner with a band pass filter with cut-off frequencies at 20 to 500 Hz, an amplifier gain of 1000 times and a common mode rejection ratio >120 dB. All data were acquired and processed using a 16-bit analog to digital converter (EMG System do Brasil Ltda®) with a sampling frequency 2 kHz. Active bipolar electrodes with a pre-amplification gain of 20x are included.

### Procedures

The participants visited the Physiology Laboratory on two different occasions, with a one-week interval between visits. Sessions were held in a silent, well-lit recording room. The participant was instructed to remain seated comfortably erect on a chair with eyes open, feet apart, shoulders relaxed and hands resting on thighs, with the head on the Frankfort plane parallel to the ground. During the procedures, the participants did not receive any visual feedback of the signals displayed on the computer.

A short training period was conducted prior to the tests to familiarize the participant with the tasks. Explanations concerning the procedures and electrode placement were given and the participant was trained to bite as hard as possible with maximum voluntary clenching (MVC) effort with a cotton role between the arches and maximum habitual intercuspation (MHI) effort with no material interposed between the teeth.

The sites for the placement of the electrodes were shaved and cleaned with a cotton ball soaked in 70% alcohol to diminish impedance between the skin and electrode. Pre-gelled, self-adhesive, bipolar, disposable, Ag/AgCl surface electrodes (MediTrace®) measuring 10 mm in diameter were positioned over the right masseter (RM), left masseter (LM), right temporal (RT) and left temporal (LT) muscles with an inter-electrode distance of 20 mm in the region of greatest tonus after the participant performed moderate intercuspation^13^. Bandage tape was used to secure the electrodes further, with care taken to avoid micro movements. A rectangular metallic electrode measuring 3 ×2 cm coated with Lectron II conductive gel (Pharmaceutical Innovations) to increase the conduction capacity and impede interference from external noise was attached to the left wrist as a ground reference.

Readings were performed under three conditions: at rest, during MVC and during MHI. In session 1 (test), three readings (10 seconds each) were performed in the rest position, followed by three readings (five seconds each) during MVC with a two-minute interval between readings. After three minutes, three readings (five seconds each) were performed during MIH with a two-minute interval between readings. The same procedures were repeated after one week in session 2 (retest).

### Data processing

**Figure 1:**
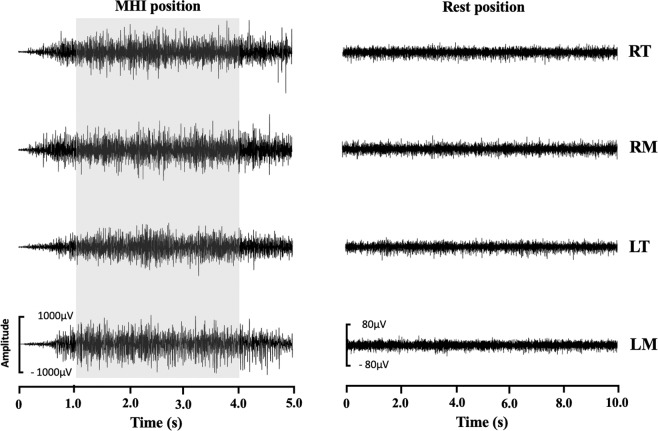
Surface electromyography signal of the right temporal (RT), right masseter (RM), left temporal (LT) and left masseter (LM) muscled during maximum habitual intercuspidation (MHI) and rest position. The grey band shows three-second period selected (Patient with Down Syndrome – mean age: 22.7 ± 6.5 years; body mass: 28.5 ± 6.8 kg/m^2^).

The sEMG signals obtained for the RT, RM, LT and LM muscles were processed using traditional linear analysis in the time (amplitude) and frequency domains as well as nonlinear analysis through the calculation of the complexity of the sEMG signal^17^. For the analysis of MVC and MIH, the first and last seconds of the five-second signals were discarded, resulting in three-second readings considered for each test. In contrast, all 10 seconds of the signals captured in the rest position were considered (Fig. 1).

The amplitude of sEMG signal was determined considering root mean square (RMS) using a 200-ms moving window. The mean RMS values of the three readings at rest and during MIH were normalized by the highest RMS values obtained during the three MVC readings (µV/µV × 100: % MVC).

The power spectral density of the sEMG signals was calculated using Welch’s averaged periodogram with a Hamming window length zero-padded to the length of 2048 points. Overlap was 50% of the window length. The mean frequency (MNF) and median frequency (MDF) of the power spectrum were calculated for each one-second window (total: three windows)^17^ and the average of the values among the three windows was used in the reliability tests.

ApEn (m, r, N) was calculated to quantify the regularity or complexity of the sEMG signals, with the embedding dimension (m) and tolerance distance (r) set to m = 2 and r = 0.20 of the standard deviation (± SD) of the data sequence and N = number of points to be analyzed^36–38^. This analysis returns a value between 0 and 2 (arbitrary units [au]), with higher values reflecting greater irregularity in the time series^36–38^.

The sEMG signals were processed using specific routines carried out in Matlab version 7.1 (The MathWorks Inc., Natick, MA, USA).

### Data analysis

The Shapiro-Wilk test was used to determine the distribution of the data and data were expressed as mean and SD. Intraclass correlation coefficients (ICCs) and the standard error of the mean (SEM) was used to verify the reliability of each sEMG measure. Intra-session reliability was determined by computing the intraclass correlation coefficient (ICC_2,1_) using only the first and second of the three measurements. Inter-day reliability (ICC_2,1_) was determined by comparing the average (3 trails) of each sEMG activity index for each muscle between the two sessions (test and retest). SEM was estimated by subtracting the ICCs value from one, taking the square root of this value, and multiplying by the SD^39^. The ICCs was interpreted using the following criteria: 0.00 to 0.39 (poor), 0.40 to 0.59 (fair), 0.60 to 0.74 (good) and 0.75 to 1.00 excellent)^40^. The SEM was used to express reliability in absolute values, with a lower SEM denoting greater reliability of the measurement, whereas a high SEM indicates a high level of error and implies the non-reproducibility of the tested values.

All data were analyzed using the Statistical Package for the Social Sciences (SPSS) Version 17.

### Ethic approval

All procedures performed in studies involving human participants were in accordance with the ethical standards of the institutional research committee (Human Research Ethics Committees of the Institute of Science and Technology– UNESP/SJC, Brazil, CEPh 2.127.141, process number CAAE 64173616.4.0000.0077) and with the 1964 Helsinki declaration and its later amendments or comparable ethical standards.

### Informed consent

Informed written consent, for adult with Down syndrome in this study, was obtained from their parent/caregiver.
